# How will we treat systemic lupus erythematosus in the next 5 years?

**DOI:** 10.1515/rir-2025-0016

**Published:** 2025-10-04

**Authors:** Jiuliang Zhao, Xinping Tian, Mengtao Li, Xiaofeng Zeng

**Affiliations:** Department of Rheumatology and Clinical Immunology, Peking Union Medical College Hospital (PUMCH), Chinese Academy of Medical Sciences & Peking Union Medical College, National Clinical Research Center for Dermatologic and Immunologic Diseases (NCRC-DID), Ministry of Science & Technology, Key Laboratory of Rheumatology and Clinical Immunology, Ministry of Education, State Key Laboratory of Complex Severe and Rare Diseases, Beijing, China

Systemic lupus erythematosus (SLE) is a complicate and highly heterogeneous systemic autoimmune disease involving multiple organ systems. It is characterized by a chronic progressive course and a tendency for remission and recurrence. Recent advancements in diagnostic methodologies and treatment modalities have markedly shifted the management paradigm of SLE from symptomatic control toward achieving sustained remission and organ damage prevention. China has a large SLE patient population with distinct clinical features compared to other ethnical groups.^[1]^ Furthermore, with continuous innovations in diagnostic and therapeutic approaches, as well as ongoing optimization of treatment strategies, it is imperative to develop an up-to-date clinical guidelines tailored to the healthcare context of China, enhancing evidence-based decision-making in clinical practice.^[2]^

Five years ago, the evidence-based guidelines for the diagnosis and treatment of SLE were endorsed by the Chinese SLE Treatment and Research Group (CSTAR) in collaboration with the Chinese Rheumatology Association (CRA). The guidelines have contributed to a significant elevation in the standard of care for SLE patients across China. Nevertheless, rapid progress in SLE research and therapy (*e.g*., chimeric antigen receptor T [CAR-T]-cell therapy) over the past five years demands updated national guidelines to align with current best practices. Therefore, under the leadership of the National Clinical Research Center for Dermatologic and Immunologic Diseases (NCRC-DID) in collaboration with the Chinese Association of Rheumatology and Immunology Physicians, the updated guidelines were recently released.^[3]^ The guidelines were developed through a systematic evaluation of the latest national and international evidence, incorporated with real-world clinical experience from China. Three major advancements in the management of SLE were introduced: new treatment targets, novel therapeutic agents, and updated management strategies.

## Defining New Treatment Targets

The 2025 guidelines emphasize the critical importance of early diagnosis and early intervention by implementing a treat-to-target (T2T) strategy. The overarching goal is to attain sustained long-term remission, thereby diminishing the flares and treatment-related adverse events. This approach ultimately aims to mitigate organ damage and reduce mortality, while significantly improving patients’ quality of life. The guidelines endorse two internationally established T2Ts: clinical remission (the DORIS, *i.e*., the definition of remission in SLE) and the lupus low disease activity state (the LLDAS).^[4,5]^ The implementation of T2T in SLE requires close monitoring (every 3–6 months) of disease activity, response to treatment and damage (both disease- and drug-related), coupled with therapy adjustments and optimization. Updated clinical evidence have demonstrated that attaining and maintaining remission and LLDAS could markedly decrease the risk of organ damage progression and is associated with improved quality of life. This signifies a paradigm shift from a conventional, empirical driven model focused on “symptom control and flare prevention” to a goal-oriented framework centered on “explicit target-setting and dynamic assessment”-representing one of the most evidence-based, practical, and forward-looking advances.

## Integrating Novel Therapeutic Agents

While reaffirming the efficacy of conventional immunosuppressive agents in the treatment of SLE, the 2025 guidelines introduced evidence-based recommendations for several novel therapeutic agents including sirolimus, biologics, and Janus kinase (JAK) inhibitors. This update highlights both global advancements and particularly significant contributions from clinical research in China.

Sirolimus inhibits the mechanistic target of rapamycin (mTOR) pathway, and leads to modulation of T-cell metabolism and activation. Real-world studies from China have demonstrated comparable efficacy with tacrolimus and mycophenolate mofetil in patients with active SLE.^[6, 7, 8]^ It has been shown to significantly reduce disease activity, facilitate glucocorticoid tapering, and exhibit potential in controlling proteinuria and relieving thrombocytopenia. Consequently, it is recommended for patients who have inadequate response to standard therapies, with specific features in helping to alleviate arthritis, skin rash, lupus nephritis (LN), thrombocytopenia, and SLE associated antiphospholipid syndrome (APS).

One of the most important advancements in treating SLE is the application of targeted biologics for active SLE patients, including belimumab, telitacicept, rituximab, obinutuzumab, and anifrolumab. A growing number of studies have shown their efficacy in remission induction and maintenance treatment of SLE. Telitacicept—a novel dual-target biologic agent developed in China—represents another innovation in lupus treatment following belimumab. By simultaneously inhibiting B lymphocyte stimulator (BLyS) and a proliferation-inducing ligand (APRIL), telitacicept has demonstrated compelling efficacy and safety in clinical studies.^[9]^ Further efficacy and safety data are anticipated from its ongoing Phase III trial.

Although anifrolumab has not yet been approved for marketing in China, it has demonstrated notable efficacy and safety in SLE patients with high type I interferon signature. Additionally, the Phase III trial (NCT04931563) has shown significant efficacy of anifrolumab in Asian patients with active SLE, successfully achieving its primary endpoint. As it is expected to be available in China in the coming years, the 2025 guidelines have included it in the recommendations.

Although evidence remains limited, JAK inhibitors (*e.g*., tofacitinib, baricitinib) have demonstrated efficacy for cutaneous and musculoskeletal manifestations of SLE in the literature, making them a potential treatment alternative for specific SLE subgroups. Owing to their widespread accessibility and affordability in China, these agents have also been included in the updated guideline.

## Optimizing Management Strategies

Compared to the 2020 guidelines, the 2025 guidelines emphasize on stratified and organ-specific management strategies, advocating for a more tailored and individualized approach. Moving far beyond the previous binary classification of renal and non-renal involvement, the updated guidelines delineate detailed, evidence-based recommendations for four important manifestations: LN, thrombocytopenia, APS, and neuropsychiatric SLE (NPSLE).

It is noteworthy that the 2025 guidelines have provided dedicated recommendations for SLE-associated immune thrombocytopenia (ITP) for the first time, outlining clear therapeutic goals with detailed first- and second-line treatment strategies. The primary treatment goal for SLE-ITP is not necessarily to normalize platelet counts, but rather to maintain a safe level that can effectively prevent bleeding events. Furthermore, a new standalone chapter is devoted to APS, which systematically integrates management strategies for SLE patients testing positive for antiphospholipid antibodies (aPL). In addition, the 2025 guidelines endorse the updated 2023 ACR/EULAR APS classification criteria and emphasize the associations between aPL positivity and various SLE-related complications, including thrombotic events, valvular disease, microangiopathy, and pregnancy morbidity.^[10]^ It also recommends personalized antithrombotic strategies tailored to different clinical scenarios.

As the ancient Warring States philosopher Xunzi wisely noted, “Though the road be long, walking will bring you there; though the task be hard, doing will make it succeed”, Through persistent and dedicated actions by consistently implementing SLE management guidelines, refining them in response to emerging evidence, and integrating them into daily clinical practice, we will light the way toward sustained remission and a better quality of life for all patients with SLE.
